# Synthesis and Evaluation of Curcuminoid Analogues as Antioxidant and Antibacterial Agents

**DOI:** 10.3390/molecules22060971

**Published:** 2017-06-11

**Authors:** Dalia R. Emam, Ahmad M. Alhajoj, Khaled M. Elattar, Nabila A. Kheder, Ahmed A. Fadda

**Affiliations:** 1 Department of Chemistry, Faculty of Science, King Khalid University, Abha 9004, Saudi Arabia; 2 Department of Pharmacology, Faculty of Pharmacy, King Khalid University, Abha 61441, Saudi Arabia; amalajoj@kku.edu.sa; 3 Department of Chemistry, Faculty of Science, Mansoura University, Mansoura 35516, Egypt; khaledelattar2@yahoo.com; 4 Department of Pharmaceutical Chemistry, Faculty of Pharmacy, King Khalid University, Abha 61441, Saudi Arabia; nabila.abdelshafy@gmail.com; 5 Department of Chemistry, Faculty of Science, Cairo University, Giza 12613, Egypt

**Keywords:** curcumin, pyrazole, isoxazole, pyrimidine, antioxidant activity, antibacterial activity

## Abstract

Diazocoupling reaction of curcumin with different diazonium salts of *p*-toluidine, 2-aminopyridine, and 4-aminoantipyrine in pyridine yielded the arylhydrazones **2a**–**c**. Arylhydrazone of *p*-toluidine reacted with urea, thiourea, and guanidine nitrate to produce 5,6-dihydropyrimidines. Further reaction of **2a** with 2,3-diaminopyrdine in sodium ethoxide solution yielded 1*H*-pyrido[2,3-*b*][1,4]diazepine derivative. *Bis*(2,5-dihydroisoxazole) is obtained from the reaction of **2a** with hydroxylamine hydrochloride, while its reactions with hydrazines afforded the respective 4,5-dihydro-1*H*-pyrazoles. The target compounds were evaluated as antioxidant and antibacterial agents. The tested compounds showed good to moderate activities compared to ascorbic acid and chloramphenicol, respectively.

## 1. Introduction

Curcumin (**1**) is considered a type of natural product compound and is naturally extracted from *Curcuma longa* L. (*Zingiberaceae*) [1]. Curcumin analogues have been reported to have variable pharmacological activities, including anticancer [2,3,4,5,6], anti-inflammatory [7], and antibacterial [8] activities, and have been used to improve wound healing [9].

Previously, Fadda et al. [10] reported the synthesis of 17 curcumin analogues with an evaluation of the compounds as antitumor agents. The obtained results of both in vitro and in vivo antitumor activity were excellent (100% dead) compared with 5-fluorouracil at a concentration of 1 M of the tested compounds. Recently, Bayomi et al. [11] have reported the synthesis and evaluation of a series of curcumin derivatives as antioxidant agents using the 2,2′-azino-*bis*(3-ethylbenzo- thiazoline-6-sulphonic acid) (ABTS) method. The compounds that bear *o*-methoxy substitution to the 4-hydroxy function exhibited significantly higher ABTS^+^-scavenging. 

On the other hand, a review discussing the antibacterial activity of curcumin against *Staphylococcus aureus* was reported by Teow et al. [12]. In addition, Moghadamtousi et al. [13] have reported on the antifungal, antibacterial, and antiviral activities of curcumin.

In connection with our studies in the chemistry of curcumin, ketones, and unsaturated ketones [14,15,16], the present work is concerned with extending attempts to synthesize a novel series of heterocyclic curcumin derivatives in order to evaluate their antioxidant and antibacterial characters.

## 2. Resultsand Discussion

### 2.1. Chemistry

**Scheme 1:**
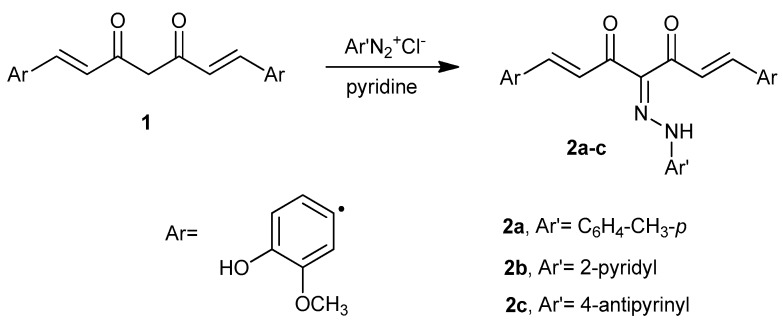
Synthesis of 1,7-*bis*(4-hydroxy-3-methoxyphenyl)-4-(2-(aryl)hydrazono)hepta-1,6-diene-3,5-diones (**2a**–**c**).

**Scheme 2:**
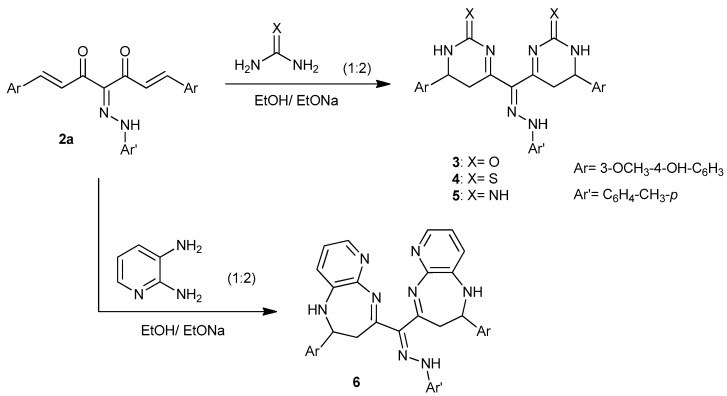
Synthesis of (pyrimidin-one/thione/imine) and diazepine derivatives.

**Scheme 3:**
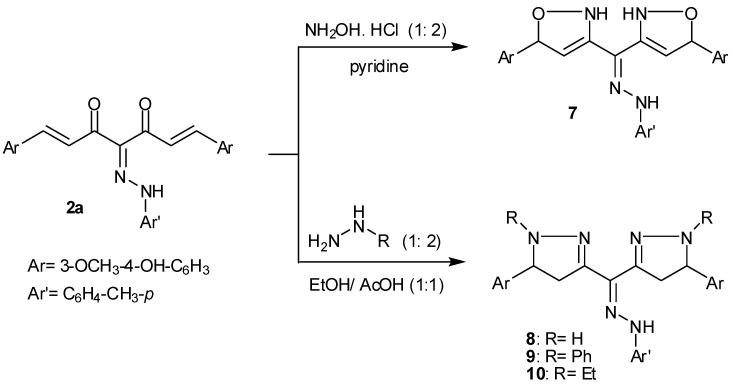
Synthesis of 2,5-dihydro-isoxazole and pyrazole derivatives.

In this work, different series of curcumin derivatives were synthesized as described in Scheme 1, Scheme 2 and Scheme 3, and their antioxidant and antibacterial activities were evaluated. 

Diazocoupling of curcumin with the appropriate aryl diazonium chlorides such as p-toluidine, 2-aminopyridine, and 4-aminoantipyrine in a pyridine solution yielded the corresponding arylhydrazone derivatives **2a**–**c** (Scheme 1). The structures of Compounds **2a**–**c** were elucidated on the basis of elemental analysis and spectral data. The IR spectra of **2a**–**c** showed absorption bands at ν = 3250, 3220, and 3218 cm^−1^, respectively, corresponding to NH-hydrazo groups. The ^1^H-NMR spectra of **2a**–**c** revealed a singlet signal in the region δ 10.83–11.03 ppm due to NH proton. The mass spectra of **2a**–**c** showed the molecular ion peaks at *m/z* 486 (M^+^, 7.31), 473 (M^+^, 16.60), and 582 (M^+^, 20.40), which are compatible with the proposed structures. 

Arylhydrazone derivative of curcumin **2a** was used as a starting material to synthesize the target compounds. Compound **2a** has two active symmetric α,β-unsaturated ketones and a 1,3-diketone moiety. Intermolecular cyclization of **2a** with *bis*-nitrogen nucleophiles (in molar ratios of 1:2), e.g., urea, thiourea, and guanidine nitrate, in refluxing sodium ethoxide and ethanol yielded the corresponding 4,4′-((2-phenylhydrazono)methylene)*bis*(6-(4-hydroxy-3-methoxy-phenyl)-5,6- dihydropyrimidin-2(1*H*)-one/thione/imine) derivatives **3**–**5** in good yields. The reaction was started through initial condensation of the amino group with the carbonyl group of the arylhydrazonocurcumin skeleton, followed by cyclization to produce the target compounds. The structures **3**–**5** were established by elemental analysis and spectral data (c.f. the experimental section). In another route, heating **2a** with 2,3-diaminopyridine in a 1:2 molar ratio worked to furnish *bis*(2-methyl-2,3-dihydro-1*H*-pyrido[2,3-*b*][1,4]diazepine) derivative **6** in good yield (Scheme 2). The ^1^H-NMR of Structure **6** revealed two singlet signals at δ 10.14 and 11.26 ppm due to NH protons, in addition to other expected protons. The mass spectrum showed a molecular ion peak at *m/z* 669 (M^+^ +1, 7.24), which is compatible with the proposed structure.

Next, our attempts were focused to prepare curcumin analogues incorporated five-membered ring systems in order to study the effect of different ring systems on biological behaviors. In this way, Compound **2a** reacted with hydroxylamine hydrochloride (in a 1:2 molar ratio) in refluxing pyridine to afford the respective 2,5-dihydro-isoxazole **7** in moderate yield. In addition, intermolecular cyclization of **2a** with hydrazine derivatives, i.e., hydrazine hydrate, phenyl hydrazine, and ethylhydrazine afforded the corresponding pyrazole derivatives **8**–**10**, respectively (Scheme 3). The proposed structures **7**–**10** are consistent with the analytical and spectral data (c.f. the experimental section).

### 2.2. Pharmacology

#### 2.2.1. DPPH Antioxidant Assay

**Figure 1:**
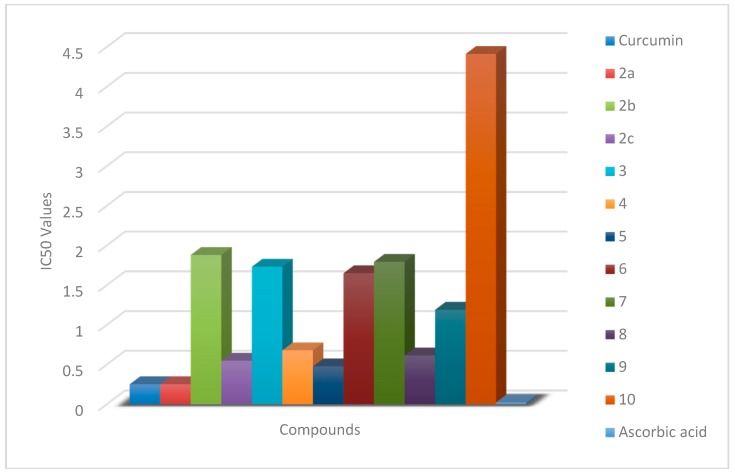
Comparison of IC_50_ values of the investigated compounds against ascorbic acid.

The target compounds were tested as antioxidant agents using a 2,2-diphenyl-1-picrylhydrazyl (DPPH) assay. The antioxidant activity was evaluated by the DPPH method at *λ* = 517 nm [17,18]. The IC_50_ values of the tested compounds revealed that the tested compounds exhibited significant antioxidant properties at a concentration 0.09 mg/mL. In other words, these compounds have the ability of electron donor to scavenge free radicals. Figure 1 showed a comparison between the values of IC_50_ of the tested compounds against ascorbic acid. 

**Figure 2:**
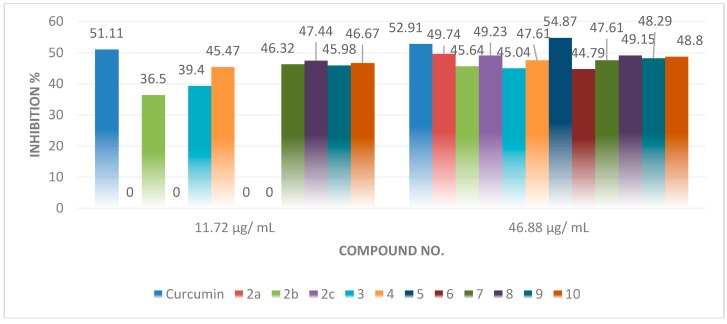
Comparison of the inhibition percentages of the tested compounds using a DPPH assay.

The IC_50_ of antioxidant activity data (Table 1 and Figure 1) indicated that curcumin and its derivative **2a** exhibited the highest antioxidant activity compared to ascorbic acid. Figure 2 shows a comparison between the inhibition percentage of the tested compounds at concentrations 11.72 and 46.88 µg/mL. The compounds at higher concentrations have higher inhibition percentages than those at low concentration. Compounds **2a**, **2c**, **5**, and **6** exhibited no antioxidant activity at low concentrations (11.72 µg/mL), while these compounds have a high activity at higher concentrations (46.88 µg/mL).

From the obtained results (Table 1), we conclude the following structure–activity relationships (SARs): (1) Heterocyclic derivatives of curcumin are less active than curcumin using low concentrations. (2) The different aryl substituents of compounds **2a**–**c** affect the IC_50_ values, the best derivative which contains a non-substituted benzene ring. (3) By comparing the structures of Compounds **3**–**5** with the obtained results, it was found that the most potent was Compound **5**, which had the lowest electronegative atom. (4) The ring size in the case of Compounds **6** and **7** has no effect on the values of the antioxidant power. (5) Compounds **8**–**10** (R = H, Ph, Et) exhibited variable IC_50_ values, of which the unsubstituted one is preferred. (6) Using higher concentrations of the tested sample increases the antioxidant power of the tested compounds (higher than 40 µg/mL).

#### 2.2.2. Antibacterial Activity

Antibacterial activity of the newly prepared target compounds against the Gram-positive (*Bacillus subtilis* and *Staphylococcus aureus*) and the Gram-negative (*Escherichia coli* and *Pseudomonas aeruginosa*) bacterial strains were examined by agar diffusion. Chloramphenicol was used as a positive control. The inhibition zones (IZ) were measured in mm. The minimum inhibitory concentration (MIC) was determined for compounds showingsignificant growth of the inhibition zone (>14 mm) using twofold serial dilution [19]. The MIC (µg/mL) and IZ (diameter) values are shown in Table 2. The inhibition zones (diameter) values mentioned in Table 2 between brackets are attributed to the tested original concentration (1 mg/mL) as a preliminary test. 

The results shown in Table 2 indicate that the majority of the tested compounds showed variable inhibitory effects on the growth of the tested Gram-positive and Gram-negative bacterial strains. In general, most of the examined compounds suggested strong antibacterial action against the Gram-positive rather than the Gram-negative bacteria.

Moreover, it showed that Compounds **4** and **10** exhibited the highest antibacterial activity compared to the results of chloramphenicol and the other tested compounds. In addition, it revealed that the pyrazole derivative of curcumin (**10**) is more potent than chloramphenicol against *B. subtilis*, while the thiopyrimidine analogue **4** is nearly equipotent to chloramphenicol against *B. subtilis*. In addition, Compounds **6**, **8**, and **9** exhibited moderate activity against *B. subtilis*, while Compounds **6**–**9** have the same effect as those of Compounds **4** and **10** on the *S. aureus* cell line. On the other hand, Compound **9** has the highest activity against Gram-negative bacteria (*E. coli*), while Compound **4** showed the highest activity against *P. aeruginosa*. Moreover, Compounds **2a**–**c** and **3** showed weak antibacterial activity compared to chloramphenicol.

By comparing the obtained results (Table 2) with the compound structures, the following SARs maybe concluded: (1) Compounds that contain six or seven membered rings are more potent than those with no heterocyclic systems. (2) The presence of high electronegative atom may decrease antibacterial activity. (3) The presence of H and phenyl substitution (Compounds **8**–**10**) decreases lipophilicity. (4) The presence of alkyl substituent (Compound **10**) enhanced antibacterial activity.

## 3. Materials and Methods

### 3.1. Chemistry

#### 3.1.1. General

All melting points were determined on a Gallenkamp electric apparatus and are considered as uncorrected. The IR spectra were recorded on a Nicolet Magna (model 550 spectrophotometers, LabX, Markham, Ontario, ON, Canada) using a potassium bromide disk. The nuclear magnetic resonance spectra were determined in DMSO on a Bruker WPSY 200 MHz spectrometer (Bruker, 15 Fortune Dr, Billerica, MA 01821, USA) using tetramethylsilane as an internal standard and the chemical shifts (δ) are in ppm. The mass spectra (energy of ionizing electron 70 electron-volt (eV) with a Varian MAT 311 spectrophotometer (Bremen, Germany). The elemental analyses for C, H, and N were satisfactory for all synthesized compounds. Curcumin (1,7-*bis*(4-hydroxy-3-methoxyphenyl)hepta-1,6-diene-3,5-dione) (**1**) was purchased from Sigma-Aldrich-08511, St. Luis, MO, USA. 1,7-*Bis*(4-hydroxy-3-methoxy-phenyl)-4-(2-(*p*-tolyl)hydrazono)hepta-1,6-diene-3,5-dione (**2a**) was prepared according to [10]. Antioxidant and antibacterial evaluation was carried out at Unit of Genetic Engineering and Biotechnology, Faculty of Science, Mansoura University.

#### 3.1.2. Synthesis of 1,7-*Bis*(4-hydroxy-3-methoxyphenyl)-4-(2-(aryl)hydrazono)hepta-1,6-diene-3,5-diones (**2a**–**c**)

**General procedure**: Preparation of the diazonium salt: 0.2 g of sodium nitrite was dissolved in 2 mL of water in an ice bath and added drop by drop to a solution of various aromatic amines, such as *p*-toluidine (0.01 mol), 2-aminopyridine (0.01 mol), and 4-aminoantipyrine (0.01 mol) in conc. HCl (3.0 mL). The formed diazonium salt solution was added drop by drop with stirring to curcumin (**1**) dissolved in pyridine (20 mL) in an ice bath. The reaction mixture was stirred for an additional two hours at 0–5 °C and left overnight in the refrigerator at 0 °C. The precipitated solid was filtered off, washed with water, dried, and recrystallized from ethanol to yield curcumin derivatives **2a**–**c**, respectively.

*1,7-Bis(4-hydroxy-3-methoxyphenyl)-4-(2-(p-tolyl)hydrazono)hepta-1,6-diene-3,5-dione* (**2a**) [10].

*1,7-Bis(4-hydroxy-3-methoxyphenyl)-4-(2-(pyridin-2-yl)hydrazono)hepta-1,6-diene-3,5-dione* (**2b**): Yield 56%; dark red powder, m.p. 176 °C; IR (KBr): ν/cm^−1^ = 3415 (OH), 3220 (NH), 1682 (C=O), 1635 (C=C). ^1^H-NMR (DMSO-*d*_6_): δ/ppm = 3.92 (s, 6H, 2 × OCH_3_), 5.84 (s, 2H, 2 × OH), 6.52 (d, 2H, *J* = 12.7 Hz, 2 × CH=CH), 6.83–7.81 (m, 10 H, Ar-H), 7.90 (d, 2H, *J* = 12.7 Hz, 2 × CH=CH),10.83 (s, 1H, NH). MS: *m/z* (%) = 473 (M^+^, 16.60), Anal. for C_26_H_23_N_3_O_6_ (473.48); Calcd. C, 65.95; H, 4.90; N, 8.87%; Found: C, 66.07; H, 4.96; N, 8.96%.

*4-(2-(1,5-Dimethyl-3-oxo-2-phenyl-2,3-dihydro-1H-pyrazol-4-yl)hydrazono)-1,7-bis(4-hydroxy-3-methoxyphenyl)hepta-1,6-diene-3,5-dione* (**2c**): Yield 68%; dark yellow powder, m.p. 184 °C; IR (KBr): ν/cm^−1^ = 3420 (OH), 3218 (NH), 1678 (C=O),1615 (C=C). ^1^H-NMR (DMSO-*d*_6_): δ/ppm = 2.26 (s, 3H, CH_3_), 3.11 (s, 3H, N-CH_3_), 3.83 (s, 6H, 2 × OCH_3_), 5.35 (s, 2H, 2 × OH), 6.67 (d, 2H, *J* = 12.2 Hz, 2 × CH=CH), 6.90–7.37 (m, 11H, Ar-H), 7.70 (d, 2H, *J* = 12.2 Hz, 2 × CH=CH), 10.83 (s, 1H, NH). MS: *m/z* (%) = 582 (M^+^, 20.40), Anal. for C_32_H_30_N_4_O_7_ (582.60); Calcd. C, 65.97; H, 5.19; N, 9.62%; Found: C, 65.86; H, 5.26; N, 9.74%.

#### 3.1.3. Synthesis of 4,4′-((2-Phenylhydrazono)methylene)*bis*(6-(4-hydroxy-3-methoxy-phenyl)-5,6-dihydropyrimidin-2(1*H*)-one/thione/imine) Derivatives (**3**–**5**)

**General procedure**: Mixtures of compound **2a** (0.01 mol) with diamines, i.e., urea (0.02 mol), thiourea (0.02 mol), or guanidine nitrite (0.02 mol), in sodium ethoxide solutions (0.02 mol sodium, in 50 mL of ethanol) were refluxed for 6–12 h. The reaction mixtures were left to cool at room temperature, poured into ice-cold water, and acidified with dilute acetic acid. The precipitated solids were filtered, dried, and recrystallized from ethanol to yield the corresponding pyrimidine derivatives **3**–**5**. 

*4,4*′*-((2-(p-Tolyl)hydrazono)methylene)bis(6-(4-hydroxy-3-methoxyphenyl)-5,6-dihydro-pyrimidin-2(1H)-one)* (**3**): Yield 41%; m.p. 235 °C; IR (KBr): ν/cm^−1^ = 3417 (OH), 3225 (NH), 1648 (C=O, amidic), 1618 (C=N). ^1^H-NMR (DMSO-*d*_6_): δ/ppm = 1.32–1.50 (m, 2H, CH_2_), 1.66–1.91 (m, 2H, CH_2_), 2.35 (s, 3H, CH_3_),3.85 (s, 6H, 2OCH_3_), 4.90 (m, 2H, 2CH), 5.41 (s, 2H, 2OH), 6.50–6.98 (m, 10H, Ar-H), 10.28 (s, 2H, 2NH), 11.23 (s, 1H, NH). MS: *m/z* (%) = 570 (M^+^, 6.43), Anal. for C_30_H_30_N_6_O_6_ (570.60): Calcd. C, 63.15; H, 5.30; N, 14.73%; Found: C, 63.27; H, 5.34; N, 14.81%.

*4,4*′*-((2-(p-Tolyl)hydrazono)methylene)bis(6-(4-hydroxy-3-methoxyphenyl)-5,6-dihydropyrimidine-2(1H)-thione)* (**4**): Yield 62%; m.p. 260 °C; IR (KBr): ν/cm^−1^ = 3415 (OH), 3235 (NH), 3223 (NH), 2935 (CH_2_), 1640 (C=N), 1278 (C=S). ^1^H-NMR (DMSO-*d*_6_): δ/ppm = 1.76–1.92 (m, 2H, CH_2_), 2.21–2.35 (m, 2H, CH_2_), 2.44 (s, 3H, CH_3_), 3.74 (s, 6H, 2OCH_3_), 4.85 (m, 2H, 2CH), 5.23 (s, 2H, 2OH), 6.34–6.87 (m, 10H, Ar-H), 10.13 (s, 2H, 2NH), 11.35 (s, 1H, NH). MS: *m/z* (%) = 602 (M^+^, 20.15), Anal. for C_30_H_30_N_6_O_4_S_2_ (602.73): Calcd. C, 59.78; H, 5.02; N, 13.94%; Found: C, 59.82; H, 5.13; N, 13.97%.

*4-(6-((6-(4-Hydroxy-2-methoxyphenyl)-2-imino-1,2,5,6-tetrahydropyrimidin-4-yl)(2-p-tolylhydrazono)methyl)-2-imino-2,3,4,5-tetrahydropyrimidin-4-yl)-2-methoxyphenol* (**5**): Yield 51%; m.p. 240 °C; IR (KBr): ν/cm^−1^ = 3422 (OH), 3200 (NH), 3180 (NH), 2915 (CH_2_), 1630 (C=N). ^1^H-NMR (DMSO-*d*_6_): δ/ppm = 1.53–1.71 (m, 2H, CH_2_), 2.01–2.24 (m, 2H, CH_2_), 2.31 (s, 3H, CH_3_), 3.85 (s, 6H, 2OCH_3_), 4.88 (m, 2H, 2CH), 5.17 (s, 2H, 2OH), 6.47–6.93 (m, 10H, Ar-H), 10.23 (s, 2H, 2NH), 11.30 (s, 2H, 2NH), 11.45 (s, 1H, NH). MS: *m/z* (%) = 569 (M^+^ +1, 12.15), Anal. for C_30_H_32_N_8_O_4_ (568.63): Calcd. C, 63.37; H, 5.67; N, 19.71%; Found: C, 63.47; H, 5.72; N, 19.78%.

#### 3.1.4. Synthesis of 4,4′-(4,4′-((2-(*p*-Tolyl)hydrazono)methylene)*bis*(2,3-dihydro-1*H*-pyrido[2,3-*b*][1,4] diazepine-4,2-diyl))*bis*(2-methoxyphenol) (**6**)

A mixture of arylhydrazone **2a** (0.01 mol) and 2,3-diaminopyridine (0.02 mol) in a sodium ethoxide solution (0.02 mol sodium, in 40 mL ethanol) was refluxed for 18 h. The reaction mixture was left to cool at room temperature, poured into ice-cold water, and acidified with dilute acetic acid. The precipitated solids were filtered, dried, and recrystallized from ethanol to yield 1*H*-pyrido[2,3-*b*][1,4]diazepine **6**. Yield 67%; m.p. 270 °C; IR (KBr): ν/cm^−1^ = 3400 (OH), 3220 (NH), 3160 (NH), 2910 (CH_2_), 1618 (C=N). ^1^H-NMR (DMSO-*d*_6_): δ/ppm = 1.74–1.90 (m, 2H, CH_2_), 2.13–2.30 (m, 2H, CH_2_), 2.42 (s, 3H, CH_3_), 3.21 (s, 2H, 2CH), 3.79 (s, 6H, 2 × OCH_3_), 5.43 (s, 2H, 2 × OH), 6.32–6.83 (m, 10H, Ar-H), 7.31–8.22 (m, 6H, Ar-H), 10.14 (s, 2H, 2NH), 11.26 (s, 1H, NH). MS: *m/z* (%) = 669 (M^+^ +1, 7.24), Anal. for C_38_H_36_N_8_O_4_ (668.74): Calcd. C, 68.25; H, 5.43; N, 16.76%; Found: C, 68.31; H, 5.51; N, 16.83%.

#### 3.1.5. Synthesis of 4,4′-(3,3′-((2-(*p*-Tolyl)hydrazono)methylene)*bis*(2,5-dihydro-isoxazole-5,3-diyl))*bis* (2-methoxyphenol) (**7**)

A mixture of arylhydrazone **2a** (0.01 mol) and hydroxylamine hydrochloride (0.02 mol) in pyridine (30 mL) was refluxed for 10 h, left to cool, and diluted with water, and the obtained solid products were crystallized from ethanol to yield isoxazole derivative **7**. Yield 53%; m.p. 200 °C; IR (KBr): ν/cm^−1^ = 3422 (OH), 3233, 3018 (NH), 1620 (C=N). ^1^H-NMR (DMSO-*d*_6_): δ/ppm = 2.31 (s, 3H, CH_3_), 3.76 (s, 6H, 2 × OCH_3_), 5.07 (d, 2H, 2CH), 5.22 (d, 2H, 2CH), 5.37 (s, 2H,2 × OH), 6.32–6.83 (dd, 4H, Ar-H), 7.31–8.22 (m, 6H, Ar-H), 10.14 (s, 2H, 2NH), 10.34 (s, 1H, NH). MS: *m/z* (%) = 518 (M^+^+2, 13.08), Anal. for C_28_H_28_N_4_O_6_ (516.55): Calcd. C, 65.11; H, 5.46; N, 10.85%; Found: C, 65.32; H, 5.54; N, 10.93%.

#### 3.1.6. Synthesis of 4,4′-(3,3′-((2-(*p*-Tolyl)hydrazono)methylene)*bis*(1-substituted/unsubstituted-4,5- dihydro-1*H*-pyrazole-5,3-diyl))*bis*(2-methoxyphenol) Derivatives (**8**–**10**)

**General procedure**: Mixtures of arylhydrazone **2a** (0.01 mol) and the appropriate hydrazine derivative, i.e., hydrazine hydrate (0.02 mol), phenyl hydrazine (0.02 mol), or ethyl hydrazine (0.02 mol), in mixtures of ethanol/glacial acetic acid (1:1) were refluxed for 4–8 h. The reaction mixtures were left to cool at room temperature and poured into ice-cold water.The precipitated solids were filtered, dried, and recrystallized from ethanol to yield *bis*-pyrazole derivatives (**8**–**10**). 

*4,4*′*-(3,3*′*-((2-(p-Tolyl)hydrazono)methylene)bis(4,5-dihydro-1H-pyrazole-5,3-diyl))bis(2-methoxyphenol)* (**8**): Yield 82%; m.p. 200 °C; IR (KBr): ν/cm^−1^ = 3400 (OH), 3212, 3052 (NH), 1605 (C=N). ^1^H-NMR (DMSO-*d*_6_): δ/ppm = 1.61–1.84 (m, 2H, CH_2_), 2.17–2.28 (m, 2H, CH_2_), 2.42 (s, 3H, CH_3_), 3.86 (s, 6H, 2 × OCH_3_), 3.96 (t, 2H, 2CH, *J* = 7 Hz), 5.67 (s, 2H, 2 × OH), 6.71–7.62 (m, 10H, Ar-H), 10.82 (s, 2H, 2NH), 11.16 (s, 1H, NH). MS: *m/z* (%) = 514 (M^+^, 25.12), Anal. for C_28_H_30_N_6_O_4_ (514.58): Calcd. C, 65.35; H, 5.88; N, 16.33%; Found: C, 65.43; H, 5.91; N, 16.46%.

*4,4*′*-(3,3*′*-((2-(p-Tolyl)hydrazono)methylene)bis(1-phenyl-4,5-dihydro-1H-pyrazole-5,3-diyl))bis(2-methoxyphenol)* (**9**): Yield 71%; m.p. 220 °C; IR (KBr): ν/cm^−1^ = 3422 (OH), 3233 (NH), 1620 (C=N). ^1^H-NMR (DMSO-*d*_6_): δ/ppm = 1.74–1.95 (m, 2H, CH_2_), 2.34–2.57 (m, 2H, CH_2_), 2.74 (s, 3H, CH_3_), 3.95 (s, 6H, 2 × OCH_3_), 4.17 (t, 2H, 2CH, *J* = 7 Hz), 5.84 (s, 2H, 2 × OH), 6.36–7.18 (m, 20H, Ar-H), 11.06 (s, 1H, NH). MS: *m/z* (%) = 666 (M^+^, 17.51), Anal. for C_40_H_38_N_6_O_4_ (666.77): Calcd. C, 72.05; H, 5.74; N, 12.60%; Found: C, 72.21; H, 5.82; N, 12.74%.

*4,4*′*-(3,3*′*-((2-(p-Tolyl)hydrazono)methylene)bis(1-ethyl-4,5-dihydro-1H-pyrazole-5,3-diyl))bis(2-methoxyphenol)* (**10**): Yield 75%; m.p. 207 °C; IR (KBr): ν/cm^−1^ = 3979 (OH), 3207 (NH), 1615 (C=N). ^1^H-NMR (DMSO-*d*_6_): δ/ppm = 1.13 (t, 6H, 2CH_3_, *J* = 7 Hz), 1.68–1.83 (m, 2H, CH_2_), 2.25–2.49 (m, 2H, CH_2_), 2.71 (q, 4H, 2CH_2_, *J* = 7.2 Hz), 2.98 (s, 3H, CH_3_), 3.78 (s, 6H, 2 × OCH_3_), 3.85 (t, 2H, 2CH, *J* = 7 Hz), 5.43 (s, 2H, 2 × OH), 6.39–6.84 (m, 10H, Ar-H), 10.08 (s, 1H, NH). MS: *m/z* (%) = 571 (M^+^ +1, 33.09). Anal. for C_32_H_38_N_6_O_4_ (570.68): Calcd. C, 67.35; H, 6.71; N, 14.73%; Found: C, 67.43; H, 6.76; N, 14.84%.

### 3.2. Pharmacology

#### 3.2.1. Antioxidant Activity

Several concentrations of each tested sample were prepared in MeOH. One milliliter of DPPH (0.012%) in MeOH was added to each concentration. The prepared sample test was left to stand in dark at room temperature for 30 min. The absorbance of each tested sample was measured at λ = 517 nm.

The inhibition percentage of the investigated compounds was calculated from Equation (1):

[(A_1_ − A_2_)/A_1_] × 100. (1)

A_1_ = control absorbance.
A_2_ = sample absorbance.

The inhibitive concentrations were obtained using the inhibition curve by drawing a correlation between the percentageof DPPH inhibition and the concentrations of each tested sample [20].

#### 3.2.2. Antibacterial Evaluation

The antibacterial activity tests were carried out according to the previously reported methods [21,22]. What man filter paper disks were obtained with a diameter size of 5.0 mm and sterilized using screw capped wide mouthed containers. Petri dishes 9 cm in diameter were kept at 150 °C, the agar media were then added to each plate, and the microorganism was seeded. The sterilized filter paper disks saturated with a solution of each tested sample in DMSO (1 mg/mL) were added to each plate in triplicates. The used tested microorganisms were different types of Gram-positive and Gram-negative bacteria, and chloramphenicol was used as a reference drug. DMSO as a solvent for the tested compound was used as a control has no effect on the bacterial growth. The plates were incubated at 37 °C for 24 h for bacterial growth. Compounds that showed significant growth inhibition zones (>14 mm) using the twofold serial dilution technique were evaluated for their MICs.

#### 3.2.3. Minimal Inhibitory Concentration (MIC) Measurement

The MIC values were measured by a determination of the optical density of bacterial cell at a wavelength (λ) of 600 nm. The experimental method that was used for the determination of MIC wascarried out according to our previously reported method [23]. 

## 4. Conclusions

A novel series of curcumin derivatives were synthesized and subjected to an antioxidant activity test using a DPPH assay and to antibacterial screening using disc diffusion. Among the newly synthesized compounds, curcumin and hydrazone derivative **2a** exhibited good activity compared to ascorbic acid, while the rest of the compounds showed moderate antioxidant activities. Results of antioxidant activity clearly demonstrated that the heterocyclic derivatives of curcumin had less activity than curcumin. Phenolic antioxidants such as curcumin compounds can inhibit free radical formation and are electron donors scavenging free radicals. The antibacterial activity of the synthesized compounds were evaluated against Gram-negative and Gram-positive bacterial strains. The results of the antibacterial evaluation revealed that the pyrazole derivative of curcumin (**10**) is more potent than chloramphenicol against *B. subtilis*. The synthesized compounds can be applied next on animals such as mice, in order to evaluate the histological and pathological diagnoses.

## Figures and Tables

**Table 1:** IC_50_ and inhibition percentage of the tested Curcumin derivatives using a 2,2-diphenyl-1-picrylhydrazyl (DPPH) assay.

Compound No.	IC_50_	Inhibition %	
		46.88 µg/mL	11.72 µg/mL
**Curcumin**	0.252	52.91	51.11
**2a**	0.253	49.74	–
**2b**	1.881	45.64	36.50
**2c**	0.548	49.23	–
**3**	1.732	45.04	39.40
**4**	0.679	47.61	45.47
**5**	0.477	54.87	–
**6**	1.650	44.79	–
**7**	1.794	47.61	46.32
**8**	0.615	49.15	47.44
**9**	1.190	48.29	45.98
**10**	4.412	48.80	46.67
**Ascorbic acid**	0.022	–	–

IC_50_ = Concentration at 50% DPPH makes scavenging.Inhibition percentage of the target compounds were calculated at concentrations 46.88 µg/mL and 11.72 µg/mL.

**Table 2:** Minimal inhibitory concentration (MIC, μg/mL) and inhibition zone (mm) of the tested compounds.

Compound No.	MIC ^a^ in μg/mL and Inhibition Zone (mm)			
	Bacteria			
	Gram-Positive Bacteria		Gram-Negative Bacteria	
	*B. subtilis*	*S. aureus*	*E. coli*	*P. aeruginosa*
**2a**	100 (15)	100 (14)	100 (16)	100 (18)
**2b**	80 (20)	80 (38)	80 (14)	80 (15)
**2c**	95 (15)	95 (18)	96 (38)	100 (18)
**3**	25 (30)	25 (21)	50 (24)	100 (15)
**4**	3.125 (38)	6.25 (34)	50 (23)	12.5 (32)
**5**	50 (25)	50 (30)	50 (18)	100 (15)
**6**	12.5 (38)	6.25 (41)	50 (19)	50 (20)
**7**	25 (30)	6.25 (39)	50 (16)	25 (35)
**8**	12.5 (40)	6.25 (40)	50 (18)	50 (19)
**9**	6.25 (44)	6.25 (41)	12.5 (37)	25 (33)
**10**	3.125 (45)	6.25 (42)	25 (35)	25 (39)
Chloramphenicol	3.125 (42)	3.125 (44)	6.25 (39)	6.25 (38)

^a^ MIC: Minimal inhibitory concentration.
